# Supplementary material for: The discovery of novel noncoding RNAs in 50 bacterial genomes
Source: Nucleic Acids Res. 2024 Apr 22;52(9):5152–65. doi: 10.1093/nar/gkae248 (PMC11109978; doi:10.1093/nar/gkae248)
Supplement: gkae248_Supplemental_Files [file gkae248_supplemental_files.zip › Narunsky 50 Genomes RNA Motifs Files.zip!/Publication/53-A_muciniphila/Figures/LRC-53-2-NC_010655_1_IGR_18_2319100_2319263.sto.pdf]

LRC-53-2-NC_010655_1_IGR_18_2319100_2319263.sto.r2r

5′-G A A C A A A C U U C C G G G G C U A U C G C C C G C A G G G A A U A U C C C U G C G G G C G C G A G A G A U U U A G C U C A G C C G C G G C A U U A C C A A G A C G A A C R C G G C G C G U A U G A U U C C G A U U U C C U C U A C R C U U G Y G G A R C A G C U G A A C U A U U U C A A A G C A G A G G U U U U U C C U G C A G G A
